# A Brain-Penetrant Stearoyl-CoA Desaturase Inhibitor Reverses α-Synuclein Toxicity

**DOI:** 10.1007/s13311-022-01199-7

**Published:** 2022-04-20

**Authors:** Silke Nuber, Chee Yeun Chung, Daniel F. Tardiff, Pascal A. Bechade, Thomas D. McCaffery, Kazuma Shimanaka, Jeonghoon Choi, Belle Chang, Waseem Raja, Esther Neves, Christopher Burke, Xin Jiang, Ping Xu, Vikram Khurana, Ulf Dettmer, Saranna Fanning, Kenneth J. Rhodes, Dennis J. Selkoe, Robert H. Scannevin

**Affiliations:** 1 Ann Romney Center for Neurologic Diseases, Department of Neurology, Brigham and Women Hospital and Harvard Medical School, 60 Fenwood Rd, MA 02115 Boston, US; 2 Yumanity Therapeutics, 40 Guest Street, Boston, MA 02135 US; 3 iNeuro Therapeutics, Cambridge, MA 02116 US; 4 Wave Life Sciences, 733 Concord Ave, Cambridge, MA 02138 US; 5 Verge Genomics, 2 Tower Pl, South San Francisco, CA 94080 US

## Abstract

Increasing evidence has shown that Parkinson’s disease (PD) impairs midbrain dopaminergic, cortical and other neuronal subtypes in large part due to the build-up of lipid- and vesicle-rich α-synuclein (αSyn) cytotoxic inclusions. We previously identified stearoyl-CoA desaturase (SCD) as a potential therapeutic target for synucleinopathies. A brain-penetrant SCD inhibitor, YTX-7739, was developed and has entered Phase 1 clinical trials. Here, we report the efficacy of YTX-7739 in reversing pathological αSyn phenotypes in various in vitro and in vivo PD models. In cell-based assays, YTX-7739 decreased αSyn-mediated neuronal death, reversed the abnormal membrane interaction of amplified E46K (“3K”) αSyn, and prevented pathological phenotypes in A53T and αSyn triplication patient-derived neurospheres, including dysregulated fatty acid profiles and pS129 αSyn accumulation. In 3K PD-like mice, YTX-7739 crossed the blood–brain barrier, decreased unsaturated fatty acids, and prevented progressive motor deficits. Both YTX-7739 treatment and decreasing SCD activity through deletion of one copy of the SCD1 gene (SKO) restored the physiological αSyn tetramer-to-monomer ratio, dopaminergic integrity, and neuronal survival in 3K αSyn mice. YTX-7739 efficiently reduced pS129 + and PK-resistant αSyn in both human wild-type αSyn and 3K mutant mice similar to the level of 3K-SKO. Together, these data provide further validation of SCD as a PD therapeutic target and YTX-7739 as a clinical candidate for treating human α-synucleinopathies.

**Supplementary Information:**

The online version contains supplementary material available at 10.1007/s13311-022-01199-7.

## Introduction

There are currently no disease-modifying therapies available for treatment of PD or related synucleinopathies such as multiple systems atrophy (MSA) and dementia with Lewy bodies (DLB). These diseases are each characterized by presence of Lewy bodies enriched in αSyn protein and are thus collectively known as synucleinopathies [1, 2]. The α-synuclein gene, *SNCA*, is mutated in rare familial forms of disease, including either missense mutations or extra copies of the wild-type (WT) gene [3, 4]. Common single-nucleotide polymorphisms in linkage with *SNCA* are among the commonest risk factors for sporadic late-onset PD.

The putative function of αSyn protein centers on membrane-dependent processes, including regulation of vesicle trafficking and dynamics [5–7]. This function of αSyn is mediated by its ability to bind to membranes, where it undergoes a conformational change upon binding from a disordered to a *α*-helical structure [8]. Conversely, αSyn interactions with lipids can directly modify membrane structure [9, 10]. While this binding interaction is important for assembly of α-synuclein into physiological multimers allowing normal vesicle dynamics [11], elevated levels of α-synuclein or mutations [12] can lead to clustering of excess αSyn monomers with vesicles in neurons [13]. Such clusters impair vesicle trafficking and dynamics [14–19]. PD patient pathology reflects these changes whereby clustered organelles and membranes are observed in developing Lewy bodies [20, 21]. The normal function of αSyn, the pathological effects on membrane-mediated functions, and the development of lipid-rich histopathological features highlight the critical relationship between αSyn and lipid biology.

This link between lipid biology and αSyn is supported by recent evidence from cell-based screens of αSyn toxicity that identified stearoyl-CoA desaturase (SCD) as a potential target for treatment of synucleinopathies. Two recent, independent studies discovered the role of SCD in mediating αSyn toxicity. In one study, small molecule inhibitors of Ole1, the yeast homolog of SCD, were found to be cytoprotective against αSyn toxicity in unbiased phenotypic compound screens [22]. In a second study, lipidomics of an αSyn yeast model revealed changes in lipids proximal to SCD activity, as well as accumulation of triacylglycerides in lipid droplets [23]. Ole1/SCD is an ER-resident fatty acid desaturase that introduces a double bond at carbon 9 of saturated CoA-conjugated fatty acids of 16 or 18 carbons in length (C16 and C18, respectively). The resulting unsaturated acyl-CoA molecules are subsequently incorporated into higher-order lipid species, including phospholipids, triacylglycerides, and cholesterol esters [24]. The level of fatty acyl chain saturation can impact membrane fluidity and thus modulate multiple membrane-dependent events, such as vesicle trafficking. Critically, SCD inhibition can be measured by quantifying individual species of fatty acids (FA) and calculating the desaturation index (FADI) for 16- or 18-carbon fatty acids. The C16 FADI is the ratio of palmitoleic (C16:1n7) to palmitic (C16:0) acids, and the C18 DI is the ratio of oleic (C18:1n9) to stearic (C18:0) acids. These indices can be monitored in cells, biofluids, and tissues and serve as a pharmacodynamic marker to assess the relationships between SCD inhibitor dose and exposures, enzyme activity, and efficacy.

Upon identification of SCD as a target, both novel and reference compounds demonstrated efficacy in enhancing survival and mitigating cellular pathologies, such as vesicle trafficking defects and triacylglyceride accumulation, from yeast to human cells [22, 23, 25]. These studies were also validated through genetic silencing of SCD in nematode models of αSyn toxicity [26]. The benefit of modulating lipid metabolism to mitigate αSyn toxicity is further corroborated by human genetics. While SCD mutations have not been directly identified, there are numerous Parkinson’s risk factors that function in lipid metabolism or related functions, such as *GBA*, *LRRK2*, *RAB7*, *VPS35*, *SYNJ1*, *ELOVL7, SCARB2,* and *SREBF-1*, which is a transcription factor that regulates expression of lipogenic genes, including SCD [27, 28].

Although not fully elucidated, the mechanism by which SCD inhibition mitigates αSyn toxicity appears to be through influencing the dynamics of αSyn interaction with vesicle membranes and structural state vs. lipidic aggregation and degeneration of dopaminergic and other brain neurons in PD-like mice [29]. Physiological tetramers (T) of αSyn are in equilibrium with monomers (M) on the membrane, and all familial PD mutations were shown to decrease this ratio [12]. If the equilibrium shifts toward monomers, the increased association with membranes can lead to clustering of vesicles and increase the risk of αSyn adopting further β-sheet containing structure found in LB-type inclusions [18 20, 21, 30]. αS excess at membranes has been modeled using an amplified version of the familial Parkinson’s disease-related E46K αSyn mutation whereby, in addition to E46K, two other cytosol-facing amino acids have been mutated to lysine [31]. This increased positive charge drives membrane interactions and is associated with reductions in T/M and an increase in cytotoxicity. SCD inhibition has also demonstrated efficacy in a 3K mouse model that recapitulates a number of relevant PD phenotypes, including a reduced T/M ratio, increased insoluble and phosphorylated-serine 129 (pS129) αSyn, proteinase K-resistant αSyn, increased lipid-rich structures, and impairments in motor function that are dopamine-responsive [13, 29].

Based on emerging links between αSyn pathology and lipid biology, which includes the demonstrated evidence for SCD inhibition in mitigating αSyn pathologies, a medicinal chemistry campaign was initiated to identify brain-penetrant SCD inhibitors as therapeutics for synucleinopathies. A lead compound, YTX-7739, was extensively profiled including detailed characterization or its pharmalogical in vitro and in both rodents and nonhuman primates [65]. Upon completion of safety toxicology studies,YTX-7739 was advanced into Phase 1 clinical trials to assess safety, tolerability, pharmacokinetics, and pharmacodynamics (https://www.trialregister.nl/trial/8258, https://www.trialregister.nl/trial/9172). Data herein demonstrate that SCD inhibition by YTX-7739 prevents the pathological effects of both wild-type and mutant human αSyn in vitro and in vivo. YTX-7739 ameliorated phenotypes in wild-type αSyn-overexpressing cultures as well as in a neurosphere model generated from patient cells harboring pathological αSyn mutations. These in vitro data were corroborated with in vivo evidence whereby YTX-7739 demonstrated efficacy in the 3K αSyn mouse model: Lipid-droplet-rich α-synuclein aggregates, neuronal fiber integrity, and motor behavioral phenotypes were improved. In all cases, efficacy was correlated with target engagement as evidenced by a reduction in fatty acid desaturation. Collectively, these data provide support for the continued evaluation of YTX-7739 as a potential disease modifying therapy for the treatment of synucleinopathies such as PD.

## Results

### YTX-7739 Mitigates αSyn Toxicity in Human Neurons

**Fig. 1:**
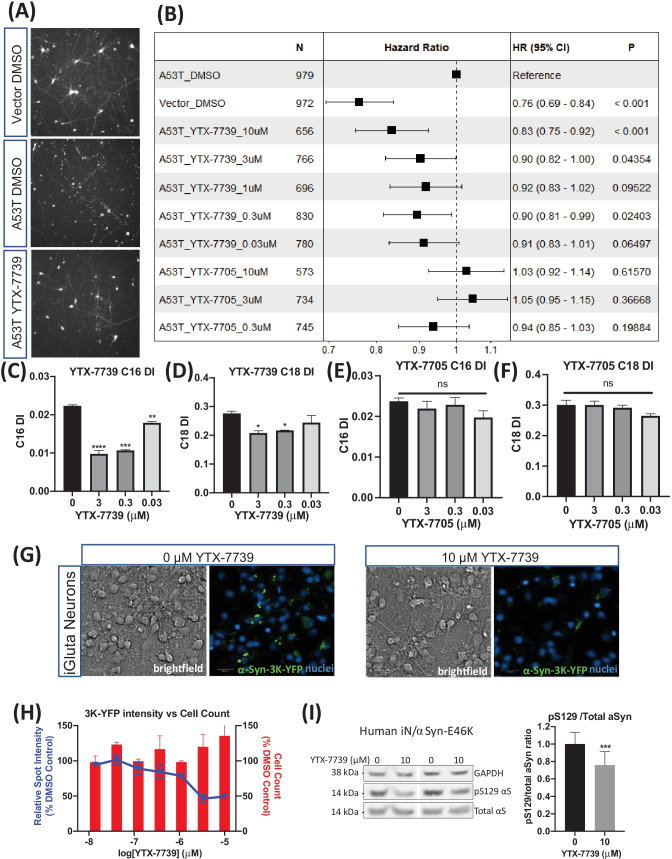
**YTX-7739 is protective in αSyn toxicity assays in human neurons.** (**A**-**F**): YTX-7739 increases survival of iCell GABA neurons overexpressing αSyn A53T. (**A**) Representative images taken from BioStation CT. Overexpression of αSyn A53T increased neuronal death, and YTX-7739 (10 μM) treatment improved neuronal survival compared to DMSO-treated neurons. (**B**) Forest plot showing the protective effect of YTX-7739 in neurons. The effect of YTX-7739 was observed in various concentrations. In contrast, YTX-7705, the inactive analog of YTX-7739, did not increase protection against αSyn A53T toxicity. (N, cell number; HR, Hazard Ratio; CI, confidence interval; P, p-value) A similar trend was observed in 5 ~ 7 independent experiments. (**C**-**F**): fatty acid desaturation index (DI) in iCell GABA neurons. Treatment of YTX-7739 for 7 days reduced desaturation of both C16 (C16:1n7/C16:0) and C18 (C18:1n9/C18:0). YTX-7705 treatment did not change fatty acid desaturation. Fatty acid desaturation index for C16 and C18 was measured after YTX-7739 and YTX-7705 treatment. Data from the duplicate were averaged (mean ± STDEV, ***; p < 0.001, **; p < 0.01, *; p < 0.05, One-way ANOVA and Dunnett’s multiple comparisons test). (**G** and **H**): YTX-7739 reduces αSyn-3K-YFP inclusions in iCell GlutaNeurons. (**G**) iCell GlutaNeurons transduced with αSyn-3K-YFP lentivirus were treated with YTX-7739 or DMSO vehicle. Brightfield and fluorescence images of the neurons treated with DMSO vehicle (indicated as 0 µM YTX-7739) and 10 µM YTX-7739 are shown. αSyn-3K-YFP was imaged in the 488 channel, while nuclei were stained with Hoechst and imaged in the 405 channel. (**H**) Quantification of the αSyn-3K-YFP inclusion intensity showed that 10 µM YTX-7739 reduces αSyn-3K-YFP inclusion in a concentration-dependent manner without affecting cell viability. All values were normalized to DMSO vehicle. Graph is means ± STDEV, n = 3. A similar trend was observed in 5 independent experiments (**I**). αSyn-E46K expressing iN cells were treated with 10 µM YTX-7739 or DMSO vehicle for 48 h and subjected to WB for total αSyn (mAb Syn1), αSyn pS129 (pAb EPY1536) and GAPDH (loading control). pS129/total αSyn ratios were calculated and normalized to DMSO vehicle (average set to 1). Two independent experiments (N = 2) were performed on 2 different days in 7 independent wells each (total n = 14). Representative Western blot and quantifications (graphs are means ± STDEV). ****, p < 0.0001. Unpaired t-test

We have previously shown that overexpression of A53T mutant αSyn in human-induced pluripotent stem cell (iPSC)-derived cortical neurons decreased neuronal survival as measured by longitudinal single cell tracking of transfected neurons [22]. This neuronal survival assay was used to examine the effect of YTX-7739 in mitigating αSyn toxicity. Neurons transfected with a plasmid encoding a red fluorescent protein (mApple) as a tracer together with either αSyn A53T or empty vector were imaged over time, and the survival of individual neurons was analyzed (Fig. S1A-C). Administering a titration of YTX-7739 (0.3 to 10 μM) after transfection significantly reduced the risk of death in neurons expressing αSyn A53T (Fig. 1A, B). In contrast, YTX-7705, a structurally similar inactive analog of YTX-7739, failed to mitigate αSyn toxicity (Fig. 1B). Efficacy correlated with YTX-7739-mediated SCD inhibition as evidenced by the decrease in both C16 (C16:1n7/C16:0) and C18 (C18:1n9/C18:0) DI compared to the DMSO control (Fig. 1C, D). The inactive YTX-7705 did not alter the DI in these neurons (Fig. 1E, F).

Next, we tested the ability of YTX-7739 to reduce αSyn-3K-YFP inclusion formation in both cell lines and human iPSC neurons. First, a human M17D neuroblastoma cell line harboring a doxycycline-inducible αSyn-3K-YFP construct was treated with a range of YTX-7739 concentrations (0.01 μM to 10 μM) or DMSO and analyzed for αSyn inclusions (Fig. S1D). The inclusions decreased in number, intensity, and spot area in the 10 μM YTX-7739-treated sample as compared to the DMSO controls. There were no effects of YTX-7739 on cell viability at any concentration. Reduction of inclusions by YTX-7739 was associated with corresponding decreases in both C16 and C18 DI (Fig. S1F). With validation in cell lines, YTX-7739 was next tested in iGluta neurons transduced with lentivirus harboring an αSyn-3K-YFP construct. Concentrations ranging from 0.12 µM to 10 μM of YTX-7739 were applied to neurons for 7 days, and αSyn inclusions were imaged and analyzed. Brightfield and fluorescent images of transduced iGluta neurons allow for visualization of viability and αSyn inclusions, respectively, in the presence of DMSO or 10 μM YTX-7739 (Fig. 1G). The αSyn inclusions decreased in number, intensity, and spot area in the 10 μM YTX-7739-treated sample in contrast to the DMSO control with no impact on cell viability (Fig. 1H), demonstrating that while inclusion intensity decreased with increasing YTX-7739 concentrations, cell viability was not affected by YTX-7739. Importantly, the reduction in αSyn inclusion formation was associated with reductions in both C16 and C18 DI at all concentrations tested when compared to DMSO controls (Fig. S1H).

We then analyzed YTX-7739 for its ability to modulate toxicity arising from the naturally occurring E46K αSyn PD mutation. A stably transformed M17D neuroblastoma line harboring mutant E46K αSyn was treated with 10, 3.3, and 1.1 μM YTX-7739 for 48 h, and pS129 αSyn and total αSyn levels were analyzed. A dose-dependent reduction in the pS129/total αSyn ratios was observed (Fig. S1G). These cell line data were then extended to human transdifferentiated neurons (neurogenin “induced neurons,” iNs) expressing E46K αSyn [25]. YTX-7739 (10 μM) or DMSO vehicle was applied for 48 h and analyzed by Western blot. This analysis demonstrated a significant reduction in the pS129/total ratio in neurons treated with YTX-7739 (Fig. 1I). Together, these data indicated that SCD inhibition by YTX-7739 reduces pathological αSyn across multiple cell types and αSyn variants.

### YTX-7739 Reversed Disease-relevant Phenotypes in PD Patient iPSC-derived Cortical Neurospheres

**Fig. 2:**
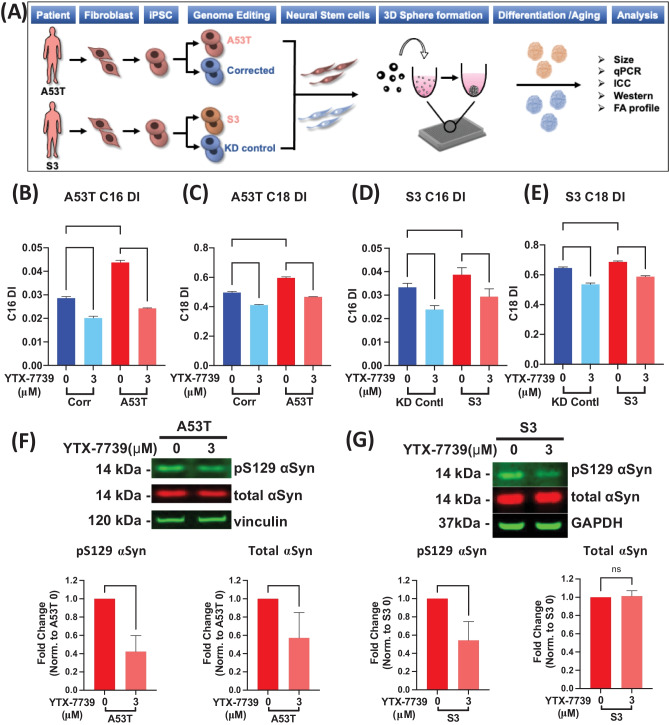
**YTX-7739 reverses disease-relevant phenotypes in patient neurospheres.** (**A**) Schematic for the generation of cortical neurospheres including iPS generation, genome editing, creation of NSCs and maturation of the spheres for analysis. (**B** and **C**) Fatty acid desaturation index (DI) for C16 (**B**)(C16:1n7/C16:0) and C18 (**C**) (C18:1n9/C18:0) on D60 A53T mutant and isogenic control (Corr) neurospheres, treated with DMSO or YTX-7739 for 2 weeks. (**D** and **E**) DI for C16 (**D**)(C16:1n7/C16:0) and C18 (**E**) (C18:1n9/C18:0) on D60 S3 αSyn triplication and isogenic control (KD control) neurospheres, treated with DMSO or YTX-7739 for 2 weeks. (**F** and **G**) Representative Western blot and quantification for pS129 and total αSyn. A53T (**F**) and S3 (**G**) cortical neurospheres were treated with 3uM YTX-7739 for 2 weeks. 80 ~ 100 neurospheres were pooled for each condition. Vinculin or GAPDH is used here as a loading control and data is normalized to their levels. Data are average of 5 (A53T) or 4 (S3) replicate experiments. Representative data are mean ± SD. Asterisks denote *p*-value by one-way ANOVA with Tukey’s test for fatty acid DI and two-tailed t-test for pS129 and total αSyn levels. (*p < 0.05, **p < 0.01, ***p < 0.001, ****p < 0.0001)

While the culturing of primary rodent and iPSC-derived neurons in two-dimensional monolayers is a common approach to study neuronal biology in vitro, the use of three-dimensional (3D) neurosphere models to more accurately reflect the microenvironment of the brain has gained traction as a discovery model [32]. We have recently developed a protocol for generating high-quality, reproducible 3D cortical neurosphere cultures [33]. Two PD patient-derived iPSC lines harboring either the A53T mutation or gene triplication (S3) in the *SNCA* gene were used to evaluate the effect of SCD inhibition by YTX-7739 on multiple disease-relevant phenotypes (Fig. 2A). Each line was paired with its isogenic CRISPR-corrected control lines: Corr (corrected) for the A53T line and KD for S3, where 2 copies of αSyn were deleted from the 4 copies of αSyn present in the parental S3 line.

Both paired iPCS lines were first differentiated into neural stem cells (NSCs) from which frozen stocks were generated. NSCs were further differentiated into cortical neurospheres and aged for 60 days in vitro (Fig. 2A). The neurospheres grew to ~ 700 μm in diameter (Fig. S2A) and contained mainly glutaminergic (vGlut2-positive) neurons as well as astrocytes (S100β-positive) (Fig. S2C-D). The neurospheres also displayed reduced levels of the neuronal precursor marker PAX6 (Fig. S2C). These cell-type protein markers were further confirmed by mRNA analyses of neuronal, progenitor, and glial markers (Fig. S2B). Overall, these data were indicative of forebrain glutamatergic neurons and glia in the neurospheres.

We observed an age-dependent increase in C16 and C18 DI in both the A53T mutant and S3 neurospheres as compared to their respective isogenic control neurospheres (Fig. 2B-E), suggesting disease-relevant αSyn mutations lead to dysregulation of fatty acid desaturation. Additionally, the A53T line exhibited decreased levels of the polyunsaturated fatty acids linoleic acid (C18:2n6) and γ-linolenic acid (C18:3n6) compared to the Corr line (Fig. S2E). In the S3 line, the level of linoleic acid (C18:2n6) was decreased compared to the KD line (Fig. S2F). Treatment of both A53T and S3 neurospheres with 3 μM YTX-7739 for two weeks restored the abnormally elevated C16 and C18 DI levels to baselines comparable to their respective isogenic control neurospheres (Fig. 2B-E). YTX-7739 also partially reversed the reduction of linoleic acid (C18:2n6) and γ-linolenic acid (C18:3n6) in the A53T neurospheres and linoleic acid (C18:2n6), in the S3 neurospheres (Fig. S2E-F). YTX-7739 also decreased the C16 and C18 DI and increased linoleic acid (C18:2n6) and γ-linolenic acid (C18:3n6) in isogenic control neurospheres (Fig. 2B-E and Fig. S2E-F). Thus, SCD inhibition by YTX-7739 normalized aberrant fatty acid profiles observed in multiple PD patient-derived lines.

In addition to aberrant fatty acid desaturation, A53T and S3 neurospheres accumulated total αSyn and pS129 αSyn levels as compared to their respective isogenic control neurospheres [33]. The abnormal accumulation of total and pS129 αSyn is a pathological hallmark observed in PD patient cells and several in vitro and in vivo model systems of PD [20]. Application of YTX-7739 in the same manner as described above reduced both the total αSyn and pS129 accumulation in A53T neurospheres (Fig. 2F). Only pS129 levels were reduced by YTX-7739 treatment in S3 patient neurospheres (Fig. 2G). These data indicate that YTX-7739 reversed disease-relevant phenotypes in PD patient iPSC-derived cortical neurospheres.

### YTX-7739 Improves Multiple Phenotypes in the 3K αSyn Mouse Model

**Fig. 3:**
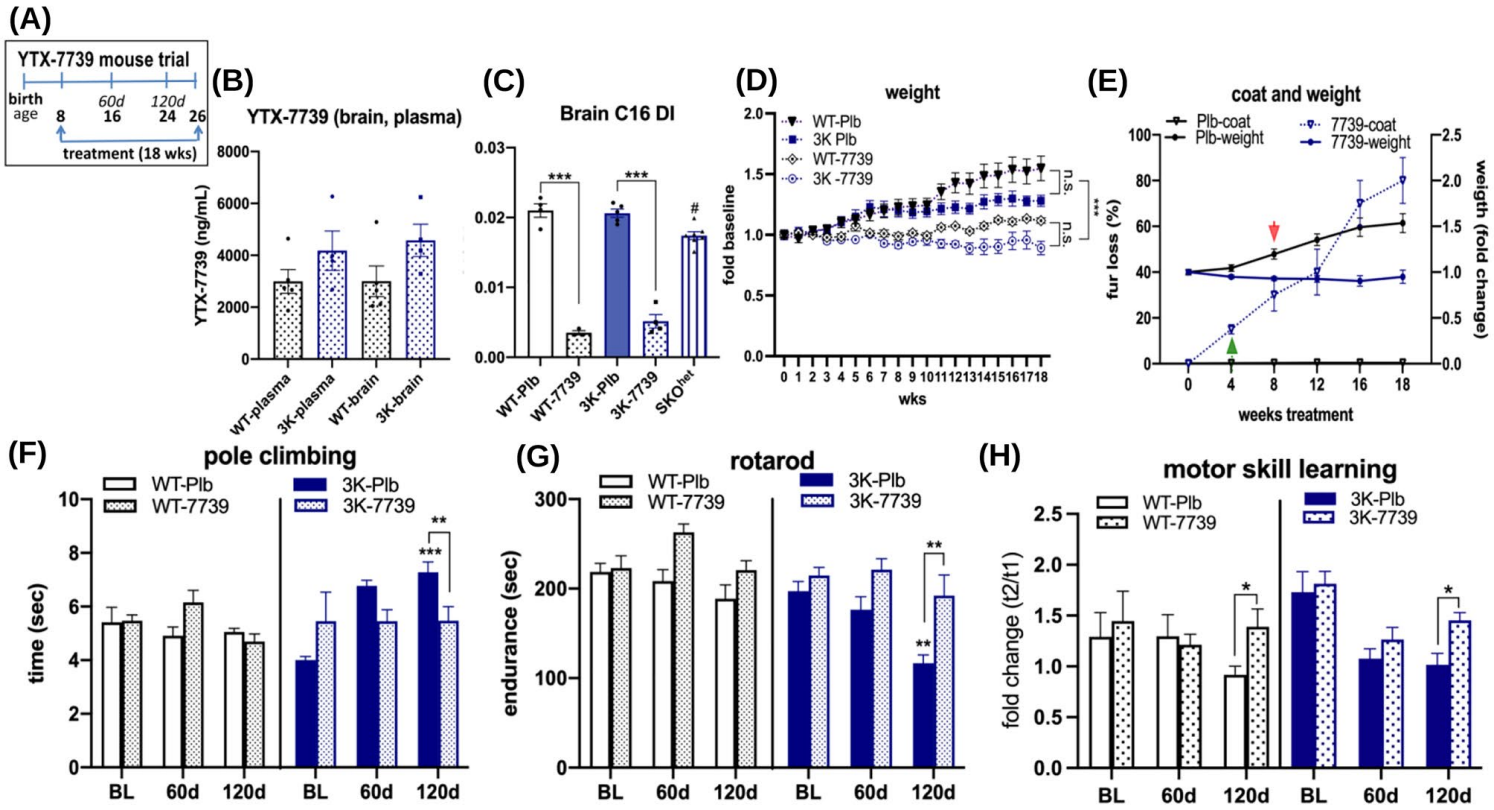
**Oral YTX-7739 (7739) bioavailability and phenotypes of placebo and treated hu WT and 3K mutant aS mice**. (**A**) Trial design orally treating mice for 18 weeks using standard rodent chow mixed with 0.2 g YTX-7739 per kg food. Phenotype assessment at baseline (BL: 8 wks), interim (60 days) and final (120 days). All mice were sacrificed at 26 wks of age. (**B**) YTX-7739 brain and plasma concentrations and (**C**) C16 desaturation indices reflecting target engagement and highly significant lowering of brain C16:1 fatty acid desaturation by YTX-7739 after 18-wk treatment. (**D**) Weight development was similar between genotypes but YTX-7739 treatment significantly lessened the weight gain vs. placebo. (**E**) Weight and fur coat in placebo and YTX-7739-treated mice. SCD inhibition resulted in fur loss, indicating efficacy of YTX-7739. Since no differences in either fur or weight were detected between genotypes, these (WT + 3K) were combined for the analyses in (**E**). Arrows indicate fur coat loss preceding stagnation in weight in YTX-7739 vs. placebo. (**F,G**) Motor performance was tested at BL, 60d, and 120d. Graphs show (**F**) changes in gait quantified by time to descend the pole and (**G**) motor performance by forced balancing on a 4–40-rpm accelerating rotarod. (**H**) Motor skill learning (at BL) and memorizing these skills (at 60d and 120d) was assessed by analyzing the fold difference of trial 2 to trial 1 of each of the first (out of 3) rotarod testing days. After 120d treatment 7739 significantly improved memorizing the skills when compared to placebo in WT and 3K mice. Data are means ± SEM; *p < 0.05, **p < 0.01, ^#^p < 0.05 (SKO vs. WT-Plb). Two-way ANOVA or (C) one-way ANOVA, post hoc Tukey

Based on the in vitro efficacy of YTX-7739 in cell lines and PD patient-derived iPSC neurons, we tested in vivo whether long-term YTX-7739 treatment of human wild-type (hu WT) αSyn and expression-matched 3K mutant αSyn transgenic mice ameliorated neuropathological and motor phenotypes. The 3K αSyn mice show progressive accumulation of pS129 and profound PD-like phenotypes, including age-related decline in gait coordination, starting as early as ~ 8–10 weeks of age through 6 months [13, 34]. Prior pharmacological assessments confirmed that YTX-7739 had favorable in vivo pharmacokinetic properties, including accessing the brain to reduce the fatty acid DI [65]. 3K mice and hu WT αSyn mice were administered YTX-7739 formulated in chow (0.2 g YTX-7739 per kg food) and fed ad libitum from 8 to 24 weeks of age (Fig. 3A). Baseline and interim motor assessments were performed until end of the study at 24 weeks. The chow intake increased slightly during this treatment period (~ 2.5–2.8 g), likely due to the higher caloric expenditure induced by the observed fur loss associated with SCD inhibition [35]. The increased uptake was maintained throughout the study, indicating a daily dose intake of approximately 15 mg/kg.

The pharmacology of YTX-7739 was confirmed by assessing both YTX-7739 concentrations as well as reductions in fatty acid desaturation. Terminal brainstem samples were used to determine compound concentrations and cortex was used to quantify the SCD substrate/product ratios. We included similar tissue pieces of heterozygous SCD1 knockout (SKO^het^) mice with 50% reduced SCD1 RNA level and SCD activity to assess effects on fatty acid desaturation [29, 36]. Eighteen weeks after initiating YTX-7739 administration, mice were euthanized, and tissue (brain, plasma) harvested. YTX-7739 achieved a brain/plasma ratio of 1:1, based on the measured concentrations (Fig. 3B), indicating favorable crossing of the blood–brain barrier (BBB) consistent to our provious studies [65]. The corresponding effects on SCD inhibition were confirmed by measuring the C16 DI in brain and plasma from all treatment groups. Treatment for 18 weeks reduced the C16 DI in hu WT and 3K brain tissue by 83% and 75%, respectively. These reductions exceeded the effects observed in the SKO^het^ mouse cortices (15 ± 2.5%) (Fig. 3C). The decrease in monounsaturated fatty acid (MUFA) by SCD inhibitors sebaceous gland atrophy and deficits in hair growth [37, 38]. At the level of SCD inhibition achieved in this study, we observed declines in fur density in YTX-7739-treated mice that were similar to the described cutaneous phenotypes of SCD1 null (SKO^hom^) mice, in which the fur loss was specifically attributed to a decrease in palmitoleic acid (16:1n7) [38]. The result also corroborated that the decrease in 16:1n7/16:0 exceeded 15–20%, since fur loss was not observed in SKO^het^ mice [36]. A two-way ANOVA revealed changes in fur loss as early as 4 weeks that preceded differences in weight, and this became significant at 8 weeks compared to placebo. Paired comparisons revealed no significant difference in weight between genotypes of either placebo or 7739 (p > 0.05; Fig. 3D); however, treatment significantly reduced weight development of the young-adult (8-wk-old) mice (Fig. 3D,E; p < 0.01). Veterinarians monitored all mice throughout the study, and the weight gain and fur loss phenotypes were not deemed to be adverse.

During the 4 months of treatment, days 60 and 120 were selected to compare motor performances in YTX-7739-treated and placebo (Plb) mice. YTX-7739-treated 3K mice consistently exhibited improved abilities in the pole climbing and the rotarod tests as compared to 3K-Plb with statistical significance achieved at day 120 due to the progressively declining performance of 3K-Plb mice (Fig. 3F, G). Acquiring and memorizing motor skills is essential for a consistent performance in the challenging rotarod test and is attributed to plasticity of motor cortex neurons [39]. This learning behavior can be measured by the improvements in endurance on the accelerating rod between the first consecutive trials of the first testing day, thereby excluding exhaustion [40]. At baseline (8 weeks), WT and 3K αSyn mice were able to quickly learn the necessary skills. After 60 and 120 days of treatment, this ability was preserved in WT and in 3K mice receiving YTX-7739 but decreased in WT-Plb and in 3K-Plb, and this became significant after 120 days (p < 0.05) (Fig. 3H). Since YTX-7739-treated WT and 3K mice displayed favorable steady-state motor performances vs Plb in all testing trials (Fig. 3G), the relative decrease in fold performance between baseline and 120 days was due to the acquisition and memorization of motor skills. The decreased motor abilities at the first testing day of each trial suggest a learning deficit in Plb and in 3K an additional decline in average motor performance of the 3-day testing period, due to the exaggerated brain synuclein pathologies vs WT [13, 29]. Thus, YTX-7739 treatments mitigated fine motor learning skills in WT and 3K and aided against the rapid motor decline in 3K mice.

**Fig. 4:**
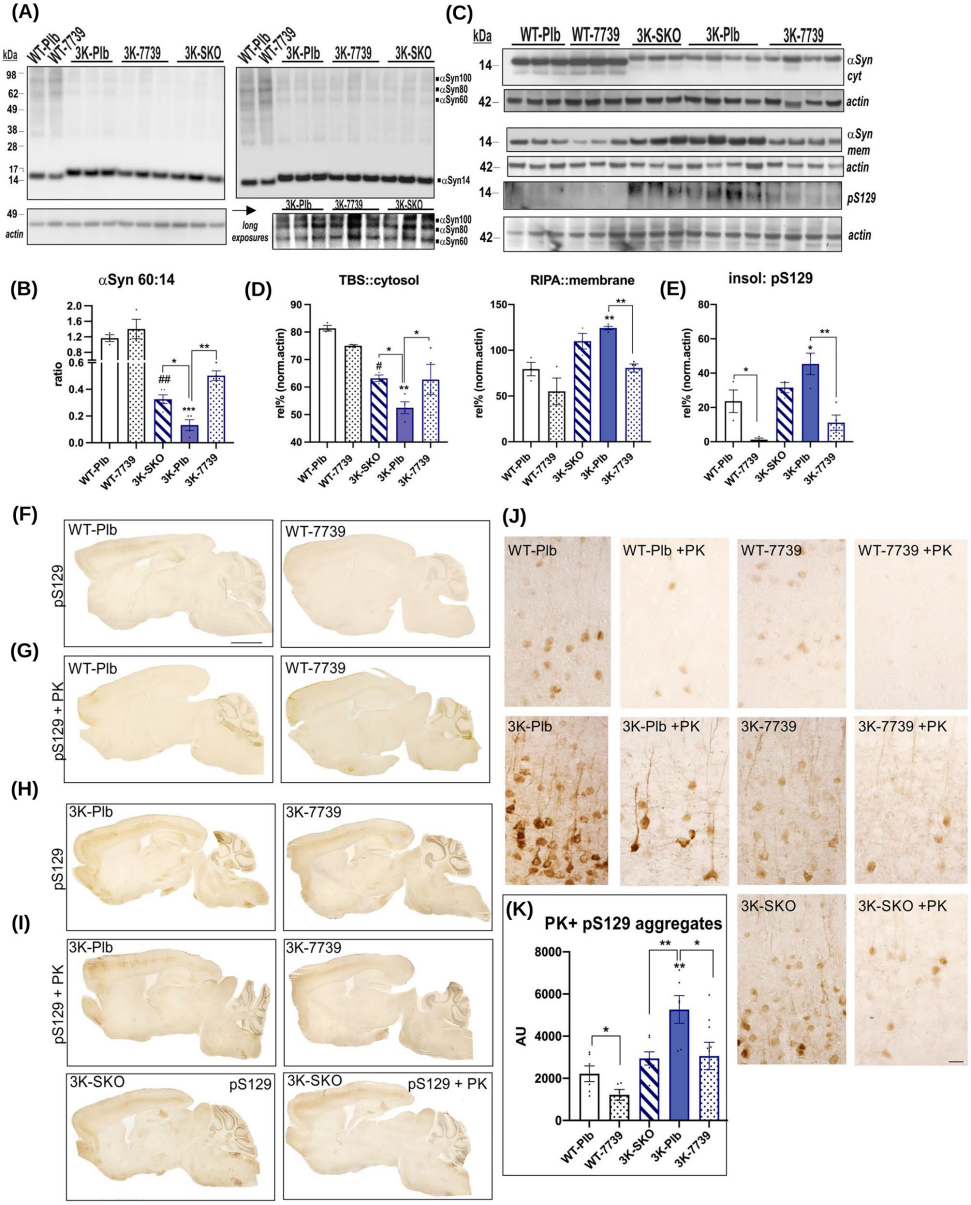
**YTX-7739 increases αSyn T/M ratio, solubility, and reduces pS129 + in 3K mice.** (**A**) Intact-cell cross-linking of αSyn using the cell-penetrant cross-linker DSG in cortical brain bits lysed with PBS/ 1% Triton-X100. Panel below: Longer exposures highlight increased αSyn tetramer signals by YTX-7739 treatment or by heterozygous crossings of 3K with SCD-KO mice (3K-SKO) mice. (**B**) Quantification of αSyn tetramer (60 kDa) to monomer (14 kDa) ratio of the corresponding blot in (**A**). (**C**) WBs of sequentially extracted cortices of hu αSyn in placebo and YTX-7739-treated WT, 3K and of 3K-SKO. Hu αSyn level of the TBS—cytosolic (cyt) fraction (upper panel) and the RIPA-membrane (mem) fraction (lower panels). Beta-actin served as loading control and for normalizing the relative expression level. (**D**) Quantification of αSyn signals. The cyt and mem fraction was developed against hu αSyn and (**E**) the detergent-insoluble fraction against serine 129 phosphorylated αSyn of the corresponding blots shown in (**C**). (**F-I**) IHC characterization of phosphorylated αSyn deposits in placebo and YTX-7739-treated WT, 3K and 3K-SKO cortices. (**F, H**) Full sagittal overview of ser129-phosphorylated αSyn in WT and 3K mouse brains and of age-matched 3K-SKO mice. (**G, I**) Adjacent sections were stained after ( +) proteinase K (PK) pre-treatment for pSer129. Note the relative decrease in pSer129 + neurons and neurites in 4-month-long treated 3K-7739 and WT-7739 brain. **(J)** Representative images of cortical sections showed PK-resistant pS129 αSyn aggregates and YTX-7739 treatment effects. (**K**) Quant. of relative proteinase K + pSer129 aggregates by optical densities (n = 10 fields of cortical layers 5 and 6 taken from 3 sections of n = 3–4 mice per cohort). Means ± SEM. *p < 0.05; **p < 0.01; (2-way ANOVA, post Tukey; genotype x treatment) ^#^p < 0.05; ^##^p < 0.01 3K-SKO vs. WT-Plb. Scale bar: 5 mm

As a first step in determining the effects of reduced SCD activity on PD-like brain pathologies, we assessed the αSyn tetramer/monomer (T/M) ratio by intact-cell cross-linking of minced cortical brain tissue. The treatment with YTX-7739 increased αSyn 60 (tetramer) and decreased the αSyn 14 (monomer) signals (Fig. 4A). Two-way ANOVA confirmed treatment (F_1,10_ = 5.065; p < 0.05) and genotype effects (F_1,10_ = 63.51, p < 0.0001). Heterozygous crossings reducing 50% of SCD1 RNA also showed a significant increase in the αSyn tetramer level in 3K-Plb vs. 3K-SKO^het^ (p < 0.05) (Fig. 4B). We previously detected tetramer abrogation associated with increased pS129 + αSyn-vesicle clusters, and such clusters are a LB-type feature of hu PD [20]. Therefore, we sequentially extracted brain cortex pieces to separate TBS-soluble from RIPA-soluble αSyn species. We observed that reducing SCD activity genetically (3K-SKO^het^ vs. 3K Plb: p < 0.05) or pharmacologically (YTX-7739) were associated with increased levels of αS 3K present in PBS-soluble fractions (p < 0.05). In accord, YTX-7739 treatments significantly decreased membrane-associated (RIPA-soluble) αSyn and (RIPA-) insoluble pS129 + (p < 0.01) (Fig. 4C, D, E). A two-way ANOVA confirmed an increase in relative solubility (TBS/RIPA ratio) by treatment (F_1,10_ = 6.051; p = 0.03) and genotype F_1,10_ = 14.87; p < 0.01). Although we only detected very limited pS129 + in the hu WT-Plb mice, this signal was also decreased in hu WT mice treated with YTX-7739 (Fig. 4D, E). A two-way ANOVA confirmed treatment (F_1,10_ = 20.28; p < 0.01) and genotype effects (F_1,10_ = 14.88; p < 0.01) without significant interaction.

To address neuropathological characteristics of pS129 + deposits, we assessed for proteinase K (PK)-resistant αSyn aggregates in cryostat sections. We digested the sections with PK and then applied a monoclonal antibody specific for phosphorylated αSyn (pSer129) (Fig. 4F-K). Treatment of 3K mice with YTX-7739 for 120 days efficiently decreased the build-up of larger-sized PK-resistant pS129 αSyn granules in multiple brain regions (see overviews in Fig. 4 F, H). The granular patterns of PK-resistant pS129 αSyn forms were relatively strong in neuronal somata of the cortical layers V and VI in 3K and therefore used for quantification (Fig. 4J). Although relatively few and small pS129 + puncta were detected in cortices of placebo hu WT αSyn mice, these were decreased by YTX-7739 treatment (two-way ANOVA: treatment, p = 0.01 and genotype, p < 0.001; Fig. 4K). Together, 120-day oral YTX-7739 treatment or deleting one allele of SCD1 decreased the C16 desaturation index to an extent that increased αSyn solubility, αSyn T/M ratio and lowered pS129 + level and PK-resistant inclusions in hu WT αSyn transgenic and 3K αSyn mutant mice.

**Fig. 5:**
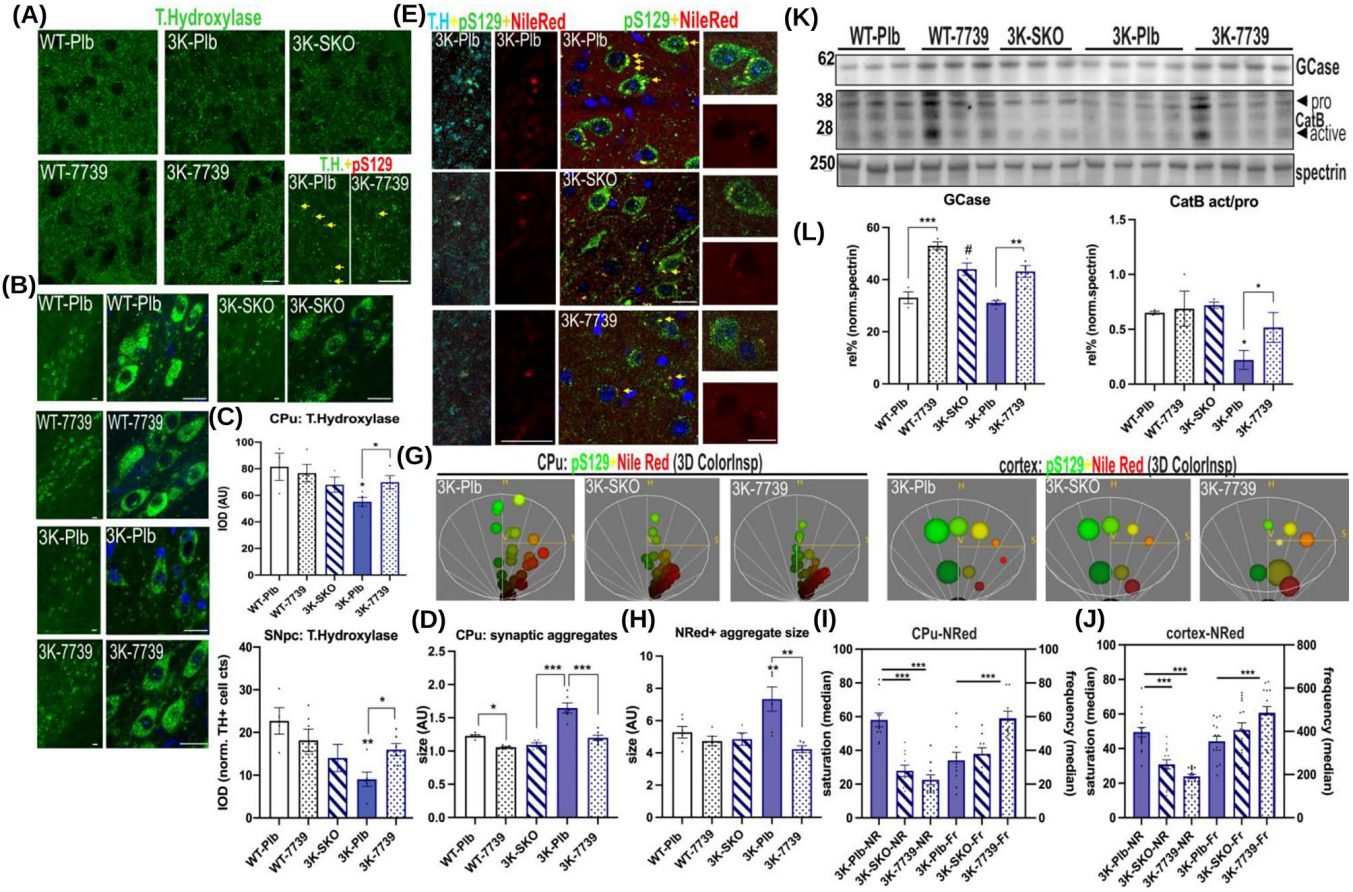
7739 treatment rescues DAergic degeneration and lysosomal integrity in 3K mice (**A**) Representative images of tyrosine hydroxylase (TH) positive striatal nerve terminals and (**B**) SNpc neurons of placebo (Plb) or YTX-7739-treated WT and 3K mice and of 3K-SKO mice. Low magnification (left panels) and high magnification (right panels) of TH + SNpc neurons. Note the relative increase in TH-immunoreactive neuropil and of nigral neurons in 7739-3K brain sections vs. Plb. (**C**) Respective graphs of the TH optical densities [10 fields of 4 sections averaged per cohort (n = 3–4 mice)]. (**D **and insets in** A**) Co-labeling with pS129 showed sizeable pS129 + puncta within TH + terminals in Plb, lowered by YTX-7739. (**E**) Confocal microscopy (merged images) of brain sections triple-labeled for the neutral lipid (droplet) marker NileRed (NRed, *red*), pS129 (*green*) + and nuclei (DAPI, *blue*). Left panels are higher magnification of TH + dopaminergic nerve termini of the dorsal striatum (caudate putamen, CPu) and right panels are representative cortical pyramidal neurons at higher magnification and highlight the reduction of sizeable lipid-rich aggregates in 3K-7739 and in 3K-SKO mouse cortex. (**G**) Colocalization of pS129 and NRed was analyzed using 3D color spectra and intensity profiles displayed in HSV (hue, saturation, volume) color model. Each axis of the 3D color spectrum represents the fluorescence intensity value of each protein (green: pS129 and red: NRed). Left panels are representative HSVs for 3K-Plb, 3K-SKO and 3K-7739 of DAergic nerve termini of the CPu and right panels of the cortical pyramidal layer. (**H**) Graph calculating the particle size (ImageJ plugin) in pyramidal cortical cell-layer co-localizing with pS129 +. (**I, J**) NRed pixels of the ROI (bright region overlapping with pS129 +) were selected with the Maxima plugin, outlined in each merged image and the median value of saturation and frequency (LUT) quantified (avg n = 10 for Plb, 3K-SKO and 3K-7739; n = 3 mice each cohort). (**K**) WB shows increase in GCase level and CatB. The CatB lower molecular weight (25–26 kDa) products represent cleavage at low pH +, consistent with lysosomal pH integrity. (**L**) Quantification of GCase and CatB signals. After blot background correction, the signal was normalized to spectrin. Data are expressed as means ± SEM; * p < 0.05, ** p < 0.01. Two-way ANOVA, post Tukey. *Scale bars*, 25 µm

Given that the 3K αSyn motor phenotypes are partly due to the degeneration of dopaminergic (DAergic) neurons of the nigrostriatal pathway [13, 34], we assessed YTX-7739 treatment effects by quantifying tyrosine hydroxylase (TH) immunopositive nerve terminals and cell bodies (Fig. 5A, B). We analyzed the dorsal striatum (caudate putamen, CPu), which is rich in projections from DAergic neurons in the substantia nigra pars compacta (SNpc). Quantification in 3K vs. 3K-YTX-7739 revealed a ~ 20 ± 6% treatment-related increase in the density of TH-immunoreactive fibers and ~ 30 ± 16% increase of TH-positive neurons in the SNpc (p < 0.05). No significant differences were observed in hu WT αSyn mice (Fig. 5C). However, both WT-7739 and 3K-7739 showed reduced pS129 + puncta colocalizing with TH + IR puncta in the Cpu (Fig. 5D). Thus, YTX-7739 treatment significantly decreased aggregates in DAergic nerve terminals of the hu WT and 3K αSyn mice.

We previously showed in 3K mice that the large-sized αSyn deposits frequently contained lipid droplets (LDs) and were in close association with late endo-lysosomal or synaptic vesicles co-localizing with pS129 +. To assess further the details of changes related to lipid deposits, we applied staining with Nile Red (NRed) to analyze the enlarged 3K deposits in DAergic terminals and pyramidal neurons of the brain cortex (Fig. 5E). NRed fluorescence allows detection of LDs due to its relative increase in intensities: LDs are brighter (more saturated) than polar (phospholipid) membranes, that display a higher frequency of the fluorophore [41]. pS129 + signals overlapping with NRed were analyzed by the 3D color-inspector (Image J plugin, NIH) (Fig. 5G). The NRed saturation (“S”) was markedly decreased by either YTX-7739 treatment or heterozygous deletion of SCD in 3K-SKO^het^ mice in striatal TH + synaptic terminals (Fig. 5G, I) and in the perinuclear area of pyramidal neurons of cortical layer V (Fig. 5J). The weighed frequency of the NRed color spectrum was increased by YTX-7739 treatment (p < 0.001) (Fig. 5I, J). These data suggest a decrease of LDs in smaller-sized aggregates [41, 42].

We previously observed the lipid-associated pS129 + αSyn colocalizing with the lysosomal membrane marker LAMP1, suggesting changes in normal lipolysis [13, 29, 34]. Therefore, we next analyzed the pattern of two key lysosomal enzymes involved in lysosomal lipid turnover, glucocerebrosidase (GCase) and cathepsin B (CatB) (Fig. 5K). Numerous studies have demonstrated that GCase level correlates with its enzymatic activity [43–46]. Western blots detected an increase in GCase signals in cortical protein extracts of YTX-7739 treated WT and 3K mice. 2-way ANOVA indicated significant treatment and genotype effects (p < 0.001). We next examined immunoreactivity for CatB, an enzyme that undergoes maturation from a high-molecular weight (HMW) form to its active low-molecular weight (LMW) form by autocleavage at low pH. LMW mature CatB aids degradation of LDs by stripping off their perilipin coat at the lysosomal membrane [47]. Of note, decreased CatB immunoreactivity and maturation have been described in iPSC-derived neurons from *GBA1*-mutant PD patients [48]. When quantifying the CatB low vs. HMW ratios, we observed a significant increase mature CatB in 3K-YTX-7739 vs. 3K-Plb brains (p < 0.05) (Fig. 5K), more similar to the pattern detected in WT-7739 (Fig. 5L). These results suggested that increases in αSyn solubility and tetramerization by YTX-7739 are associated with improved lysosomal integrity and thereby lysosomal lipid turnover.

## Discussion

The development of a disease-modifying therapy for PD and related synucleinopathies has proven to be a major challenge. SCD has been recently identified as a potential target for synucleinopathies in multiple and distinct model systems [22, 23, 25, 29, 49]. Subsequent to the discovery of SCD as a potential therapeutic target, YTX-7739 was identified as a brain-penetrant SCD inhibitor and is currently being evaluated in Phase 1 PD clinical trials (https://www.trialregister.nl/trial/8258, https://www.trialregister.nl/trial/9172) (see also [65]). Here, we demonstrate that SCD inhibition by YTX-7739 prevented the pathological effects of both WT and mutant αSyn in culture and in vivo. YTX-7739 ameliorated phenotypes in in vitro A53T αSyn overexpression assays, as well as in a neurosphere model generated from patient-derived iPSCs bearing αSyn mutations. These cell culture data were corroborated with in vivo evidence that YTX-7739 was efficacious in the 3K αSyn mouse model of PD, in that both neuronal αSyn cytopathologies and motor behavioral phenotypes were reversed.

The in vitro αSyn toxicity assays used to examine the efficacy of YTX-7739 recapitulated diverse aspects of αSyn toxicity, whether using overexpression or endogenous native expression of PD-causing mutations or amplifications thereof. The longitudinal survival assay in iGABA neurons measured general neuronal health after acute overexpression of A53T αSyn via transient transfection, while the 3K-αSyn-YFP inclusion assay reflected the abnormal membrane interaction of an amplified familial PD E46K mutation. The PD patient iPSC-derived neurosphere model, however, relied on endogenous αSyn expression in the familial PD patient neurons, with comparison to isogenic mutation-corrected controls, providing additional disease relevance as compared to the overexpression models. Both the A53T and αSyn triplication (S3) neurospheres at day 60 recapitulated the key pathological features of accumulation of total αSyn and pS129 αSyn. In addition, both the A53T and S3 neurospheres exhibited altered fatty acid profiles compared to their respective isogenic mutation and gene amplification corrected controls, corroborating the hypothesis that lipid dysregulation may be a key cytopathological feature in synucleinopathies [7]. The effect of YTX-7739 on these phenotypes was largely consistent in reversing these phenotypes between two patient lines but with a subtle difference. YTX-7739 normalized pS129 αSyn in the S3 neurospheres without lowering αSyn levels, whereas pS129 αSyn and αSyn levels were both lowered in the A53T neurospheres. The elevated αSyn levels in the S3 line may be more challenging to normalize at the cellular level due to the strong genetic driver of four copies of the *SNCA* gene. The pS129 αSyn levels in the S3 line are a secondary response to the elevated αSyn which might be more amenable to modulation. Similarly, in the A53T line, both pS129 αSyn and αSyn accumulation are secondary responses to the A53T mutation of the SNCA gene. It is possible that earlier and/or longer application of YTX-7739 might have an effect on the αSyn levels in the S3 line.

In 3K αSyn PD-like mice, pharmacological SCD inhibition via YTX-7739 decreased cellular C16 desaturation index by ~ 80% in brain, which in turn markedly reduced the excess of αSyn membrane-bound monomers. The 3K αSyn mutation shifts a physiological form of αSyn [12], the helical tetramer [50], toward excess free monomers, leading to multiple features of PD [13, 34]. Thus, the current study adds to accumulating evidence that stabilizing a portion of αSyn tetramers by decreasing monounsaturated FA levels protects from cytotoxicity in neuronal culture [22, 23, 25] and in neurons of the 3K αSyn mutant mouse [29]. The YTX-7739-mediated increases of TH + midbrain neurons and projection fibers were associated with improved pole climbing, which we previously showed was a L-DOPA responsive motor impairment in this model [13]. In addition, YTX-7739 efficiently prevented the rapid motor decline on the accelerating rotarod by improving the learning and memorization of necessary motor skills, a process based on the plasticity of cortical neurons [39]. In hu WT αSyn and 3K mutant αSyn mice, cortical neurons accumulate proteinase K-resistant pS129 + inclusions that are observed as relatively small dots in WT but develop to large, lipid- and vesicle-rich aggregates in 3K; similar inclusions are described for αSyn cytopathology preceding cell death in PD [51–54]. We find that shifting the excess membrane-bound αSyn monomers toward soluble tetramers by either genetic ablation of one SCD allele or inhibiting SCD with YTX-7739 could revert αSyn aggregation pathology. Specifically, YTX-7739 reduced LDs in proximity to pS129 puncta at TH + nerve terminals and perinuclear lysosome-rich clusters. YTX-7739 normalized lysosomal markers (LAMP1, GCase, and cathepsin B), which we had previously observed to associate with the pathological αSyn aggregates in 3K mouse brain [55]. In addition, LDs appear to be part of LBs [20, 56–58] and can contribute to the PK resistance of αSyn inclusions [59, 60]. Prolonged YTX-7739 treatment prevented the build-up of PK-resistant granule-like dots in hu WT mice and reduced the larger and LD-rich inclusions of 3K mice. Therefore, YTX-7739 improvement of dysfunctional lipid metabolism in all of our models is in accord with increasing evidence for its impact in PD [7, 61].

In conclusion, YTX-7739 potently attenuates αSyn-mediated cytopathology in cultured neurons and reduces lipid-rich brain aggregates in transgenic mice, thereby improving their motor and learning deficits. Our data demonstrating broad protection against diverse αSyn-dependent phenotypes by SCD inhibition, along with the established links among αSyn, lipids, and PD, support the continued development of YTX-7739 as a therapeutic agent for synucleinopathies.

## Materials and Methods

### Longitudinal Survival Assay in iCell GABA Neurons

#### Neuron Plating, Culture, and Transfection

iPSC-derived human cortical neurons were purchased from Fujifilm Cellular Dynamics (iCell GABA neurons, cat# R1013) and plated onto the Corning BioCoat Poly-D-Lysine 96 well plate (cat# 3842) which has been coated with Laminin (Sigma L2020, 1:300 dilution in water) a day before of neuron plating. Approximately 55,000 neurons were plated per well. Whole medium (200ul/well) was changed the next day, and then, half amount of medium was changed every 3–4 days. BDNF (50 ng/ml) was included in the medium to promote basal neuronal health. Seven days after plating, red fluorescence tracer plasmid (pSF-MAP2-mApple) and empty or αSyn-A53T mutant plasmid were introduced into neurons using Lipofectamine 2000 (Thermo Fisher) according to the manufacturer’s guide with slight modification as described previously [22].

#### Automated Time-lapse Live Imaging and Survival Analysis

After transfection, the plate was inserted into BioStation CT (Nikon) which consists of cell incubator and automated imaging system. Neurons which express red fluorescence in six technical replicates were automatically imaged with 10 × objective lens every 12 h for 12 days. In each well, 4 × 4 tiled images were captured. Individual neurons were tracked by monitoring red fluorescence with the CL-Quant software (Nikon) and automated algorithm developed by Nikon. The risk of neuronal death was determined over time, and Cox proportional hazard analysis was used to estimate cumulative risk of death, or hazard ratio (HR). An open-source R package (https://cran.rproject.org/web/packages/survival/index.html) was used to perform statistical analysis and to generate Forest plot.

#### Fatty Acid Desaturation (FADI) Measurement

Corning BioCoat Poly-D-Lysine 12-well plate (cat# 354,470) was coated with Laminin (Sigma L2020, 1:300 dilution in water) overnight, and iCell GABA neurons (Fujifilm Cellular Dynamics, cag# R1013) were plated with the density of ~ 600,000 cells/well. At DIV7, half amount of culture media was removed and new media containing YTX-7739 or YTX-7705 were added (final DMSO concentration, 0.05%). Another half media/compound change was done at DIV10. At DIV14, neurons were washed with PBS and scraped from plates with ice cold 80% methanol. The samples were stored in -80ºC and analyzed by OmegaQuant for fatty acid profiling. Desaturation index (DI) was evaluated by calculating C16:1n7/C16:0 and C18:1n9/C18:0.

### αSyn-3K-YFP Assay in M17D Stable Line and iCell GlutaNeuron

#### Plating and Maintenance

The α-Synuclein-3K-YFP M17D stable cell line was generated by transduction of naïve M17D cells (ATCC, Manassas, VA, #CRL-2267) with lentiviral particles generated from the lentiviral vector pLIX402 with an insertion of the αSyn-3K-YFP sequence**.** The line was maintained in 1:1 F-12:EMEM supplemented with 10% tetracycline-tested fetal bovine serum with puromycin selection (2 μg/mL). Cells were maintained in a 37° incubator with 5% CO_2_. iCell GlutaNeurons (Fujifilm Cellular Dynamics, Inc. #R1034) were plated and maintained according to manufacturer’s instructions with the exception of the use of PEI (Poly(ethyleneimine) solution), Sigma #181,978-100 g) instead of PDL (poly-d-lysine). Overexpression of αSyn-3K-YFP was achieved through transduction with lentiviral particles generated from the lentiviral vector pLV with an insertion of the αSyn-3K-YFP sequence**.** Neurons were transduced with lentiviral particles for approximately 24 h. Cells were maintained in a 37° incubator with 5% CO_2_.

#### YTX-7739 Compound Treatment

YTX-7739 treatment of iCell GlutaNeurons was started 24 h after transduction, and compound concentrations were maintained during media changes. Compound treatment was maintained for 7–8 days. For the αSyn-3K M17D stable cell line, compound treatment was commenced 24 h post-plating. Two days after the start of compound treatment, expression of αSyn-3K-YFP was induced by the additional of doxycycline. YTX-7739 compound concentration was maintained during the increase in media volume. Total number of days of compound treatment in the stable M17D cells was 3 days.

#### Image Acquisition

Images were acquired on the Operetta CLS high-content analysis system (PerkinElmer, Waltham, MA) using the brightfield, Hoechst 33,342, AlexaFluor 488 and/or AlexaFluor 647 channels. For the M17D stable cells, cells were stained with Hoechst (Thermo Fisher Scientific, Waltham, MA, #62,249) and plasma membrane with CellMask DeepRed Plasma Membrane Stain (Thermo Fisher Scientific, Waltham, MA, #C10046). For the iCell GlutaNeurons, neurons were stained with Hoechst. Images were analyzed using the integrated Harmony analysis software package to detect nuclei, cells and spots.

#### FADI Measurements

For M17D stable cells, samples for FADI measurements were grown in either PDL-coated 6 well plates (Corning, Corning NY, #356,413) or 12 well plates (Corning, Corning NY, #356,470) following the same compound treatment schedule as above. After the 24 h post-doxycycline induction, cells were washed 2X with cold PBS (2–3 mL) and then harvested by scraping the cells in each well with 750 µL 80% methanol (cold) followed by 750 µL 80% methanol rinse of each well to recover any remaining cells. The two methanol steps for each well were combined, centrifuged at 300 × *g* for 5 min, and then stored at -80 °C. The samples were shipped on dry ice to OmegaQuant, LLC (Sioux Falls, SD) and processed for full fatty acid profiles using gas chromatography with flame ionization detection (GC-FID) using their standard methods. Fatty acid composition was expressed as a percent of total quantified fatty acids. The fatty acid desaturation index for palmitoleic acid was calculated as the ratio between C16:1n7 to C16:0 and for oleic acid using the ratio between C18:1n9 to C18:0 in Microsoft Excel and plotted using GraphPad Prism software. For iCell GlutaNeurons, samples for fatty acid desaturation index measurements were grown in 12 well TC-treated plates (Corning, Corning NY, #3512) that was coated as described above and following the same schedule also as described above. Samples were harvested in the same manner as the M17D stable cells.

### p129S Measurement in αSyn E46K Expressing M17D and iN Cell Lines

#### Plating and Maintenance

M17D/αSyn-E46K cells and human iNs expressing αSyn E46K were cultured and treated as published [25].

#### Antibodies

Primary antibodies used were monoclonal antibody (mAb) Syn1 (BD Biosciences; 1:1000 in WB), mAb 6C5 to GAPDH (Santa Cruz; 1:1000 in WB), and polyclonal antibody (pAb) to αSyn pS129 EP1536Y (Abcam, 1:1000 in WB). Secondary antibodies used were LiCor IRDye 926–68,076, 926–32,212, and 926–32,211, 1:10,000 for WB. pS129/total αSyn Western blots were developed for total and pS129 αSyn on the same membrane (total αSyn in red channel, IRDye 926–32,212; pS129 αSyn in green channel, 926–32,211).

#### Sample Preparation pS129/Total αSyn Western Blot

Cells were washed with PBS once and lysed immediately in 1X NuPAGE LDS sample buffer by sonication (Sonic Dismembrator model 300; microtip setting = 40; 15 s) and boiling for 10 min.

##### Immunoblotting

Samples were electrophoresed on NuPAGE 4–12% Bis–Tris gels with NuPAGE MES-SDS running buffer and SeeBlue Plus2 molecular weight marker (all by Invitrogen) at 120 V and transferred in the iBlot 2 system (Invitrogen) to PVDF membranes (iBlot 2 PVDF regular stacks; IB401031). Membranes were fixed for 10 min in 4% paraformaldehyde (in PBS). Membranes were blocked in blocking buffer (TBS blocking buffer, LiCor 927–60,001) for 1 h and incubated in primary antibody in TBST Licor blocking buffer overnight at 4 °C. Membranes were washed 3 × 10 min in TBST. Secondary antibodies were prepared in the blocking buffer and incubated for 1 h at RT. Membranes were washed 3 × 10 min in TBST and scanned (Odyssey CLx, Li-Cor).

### Neurosphere Generation from PD Patient-derived iPSCs

#### iPSC Generation, Editing and Maintenance

All cells were maintained in a cell culture incubator at 37 °C with 5% CO2. The induced Pluripotent stem cells (iPSC) were generated from fibroblasts derived from a skin biopsy of Parkinson’s disease patients with either an A53 > T mutation (A53T) or gene locus triplication (S3C) in *SNCA*, the gene which encodes αSyn). Reprogramming into iPSC was performed at Harvard stem cell core using a non-integrative (mRNA) strategy. The iPSCs were expanded and maintained in mTESR media from stem cell technology. The disease-causing A53T mutation or *SNCA* triplication was corrected in house using the CRISPR-Cas9 system to generate isogenic control pairs. Details of the mutation correction for these lines is described [33]. Multiple clones were evaluated and confirmed to have a normal karyotype.

#### Generation of Neural Stem/Progenitor Cells (NSC)

On the day zero of differentiation, the iPSCs (both disease and corrected isogenic control) were lifted from surface as a single cell suspension using Accutase. After resuspension of iPSC, the Accutase was diluted using DPBS followed by spinning down to a pellet. The supernatant was aspirated, and the iPSC were re-plated onto Matrigel-coated 6 well plates in Neural Induction Media (NIM) with Rock Inhibitor. On day one when the culture should be approximately 20% confluent, the media were replaced with NIM without Rock inhibitor followed by replenishing the media every other day and any non-neural or unwanted colonies were manually scraped away. On day seven of differentiation, the cells were suspended into single cell suspension using Accutase and DPBS, and re-plated on a Matrigel coated 6 well plate in NIM with Rock inhibitor at ~ 2 × 10^5 cells/cm2. The following day, the media were switched to Neural Expansion Media (NEM). The NSC were examined under the microscope for unwanted neural crest cells, i.e., larger and flatter cells. The neural crest cells were removed by Accutase treatment. Briefly, the cells were washed with DBPS, followed by adding Accutase to the well, and incubated for two minutes at 37 °C, and then transferred to a microscope at room temperature, and plates were observed under the microscope and kept in Accutase until all the flat cells lifted. Once all the neural crest cells were lifted, Accutase was aspirated and the culture was gently rinsed with DPBS three times. The remaining cells were suspended into a single cell suspension in NEM and re-plated at ~ 2 × 10^5 cell/cm2 on a Matrigel-coated plate with rock inhibitor. The following day, media were replaced with fresh NEM without rock inhibitor and the cultures were examined for neural crest cells for next few days. If neural crest cells were observed, the Accutase cleaning steps were performed as described above. After achieving the pure population of NSC, the NSC were transfer onto Poly-L-ornithine/laminin, or Poly-D-lysine/Laminin surfaces. The NSCs were expanded and frozen in Synth-a-freeze Cryopreservation Medium.

#### Generation of Neurospheres

NSCs were maintained and expanded in NEM. To generate neurospheres, the NSC were lifted from the surface as a single cell suspension using Accutase. After washing out Accutase, the NSC were resuspended in NEM and strained through 40-micron filter. The density of the cell suspension was adjusted to plate 20,000 cells per well. The cell suspension was added to 384 well ultra-low adhesion spheroid plates, leaving the outermost rows and columns filled with sterile water. After loading cells, the spheroids plates were centrifuged at 300 g for 5 min to aggregate the cell in the bottom of the well and to remove any air bubbles in the media. The neurospheres were formed overnight in each well. Around 80% of the NEM was aspirated and replaced with differentiation media to initiate differentiation. The differentiation media were changed every other day. After ten days of differentiation, the differentiation media were replaced by maturation media (iCell Complete Maintenance media). The media were changed bi-weekly for the next three weeks and then once a week after day 30. The total media volume was approximately 80 µl per well in a 384 well plate and in each media change 60 µl was aspirated and same volume was added back to each well of 384 well plate. The neurospheres were harvested at different time points using wide orifice pipette tips for characterization. The desired number of spheres were added to microfuge tubes, washed with DPBS, and proceeded to storage or processed and fixed.

#### Compound Treatment

Forty-five-day-old neurospheres from both patient-derived and isogenic control lines were treated with 3µM YTX-7739 along with DMSO as a vehicle. The final concentration of DMSO in the media was 0.03%. The spheres were treated twice a week and harvested after two weeks of treatment. The spheres were harvested and pooled from multiples wells of 384 well plates for different assays. The spheres were harvested and washed once with DPBS and processed for the appropriate assay.

#### RT-qPCR

Neurospheres were washed once with DPBS and processed for RNA extraction using RNeasy Plus Mini Kit (from Qiagen) following the manufacturer’s protocol. The amount of RNA was quantified using a nanodrop. The extracted RNA was processed using qScript cDNA Supermix (from Quanta Biosciences) and the Mastercycler ep to generate cDNA using RT-PCR, with the starting 20 ng/ul RNA concentration for all the samples. Gene expression was then analyzed with qPCR using the Taqman Fast Advanced Master Mix (from Thermofisher), Taqman Gene Expression Probes, and the Step One Plus Real-Time PCR System. Genes assayed: *Pax6, MAP2, S100β, VGLUT2, Synapsin I, GAD2*, along with housekeeping gene *GAPDH*. Taqman assays: MAP2: Hs00258900_m1; VGLUT2: Hs00220439_m1; GAD2/65 (for GABAergic Neurons): Hs00609534_m1; PAX6: Hs01088114_m1; Synapsin I: Hs00199577_m1; S100B: Hs00902901_m1; GAPDH: Hs03929097_g1.

#### Immunohistochemistry

Neurospheres were harvested as mentioned above and fixed in 4% PFA overnight. PFA was washed out, and neurospheres were stored at 4 °C in PBS with 0.05% sodium azide. The neurospheres were cryosectioned onto 14 ~ 20 µm on glass slides (by Histoserv Inc). The sections were stained in house using an already published protocol [33]. Briefly, the sections were washed once with PBS and permeabilized for 30 min in PBS containing 0.3% Triton-X100 called PBST and blocked in 10% v/v normal goat serum or horse serum in PBST solution for 1 h. The blocking solution was replaced with primary antibodies solution and incubated overnight at 4 °C in PBST containing 5% normal goat serum or horse serum. Sections then received three 15 min washes in PBST containing 5% normal goat serum or horse serum. Secondary antibodies were prepared in PBST containing 5% goat serum and Hoechst (for nuclear immunoreactivity), and sections were incubated in secondary antibodies for 2 h at room temperature before washing as before. Sections were then cover slipped in Fluoromount-G mounting medium and the edges sealed with nail polish. Slides were imaged using a Nikon confocal microscope.

#### Western Blotting

Approximately 60 to 80 neurospheres were harvested and stored at -80C as mentioned above. The neurospheres were homogenized in lysis buffer (20 mM HEPES, 150 mM NaCl, 10% Glycerol, 1 mM EGTA, 1.5 mM MgCl2, 1% Triton X-100, + protease (Sigma P8340) and phosphatase (P2850, P5726) inhibitors) using a pestle. After homogenization, the samples were incubated on ice for 20 min and subjected to two freeze–thaw cycles. Following freeze–thaw, samples were centrifuged at 14,000 g for 20 min at 4 °C to sediment insoluble material. Supernatant protein was harvested and measured using the BCA method (Thermofisher 23,225). Forty micrograms of total protein was separated on 4–12% SDS PAGE gels (Invitrogen WG1401A) and dry transferred to 0.22uM PVDF membranes. The membrane was fixed in 1% formaldehyde for 40 min prior to blocking for 30 min at room temperature (LiCor Odessey blocking buffer 1:1 in TBS). The membrane was incubated in Primary antibodies overnight at 4 °C with gentle rocking. Following primary antibody incubation, blots were washed three times for 15 min each in TBS solution containing 0.01% tween20. The membrane was then incubated in secondary antibodies at room temperature for 2 h followed by three washes in TBS and 0.01% tween20 solution. After the washes, the blots were imaged on a Li-Cor Odyssey CLX and analyzed on LiCor ImageStudio software.

##### Fatty Acid Desaturation Index (FADI) Analysis

Neurospheres were harvested as mentioned above, washed once with DPBS and resuspended in 500 µl ice cold 80% methanol and stored at -80C until ready to process. The samples were shipped (on dry ice) to OmegaQuant LLC and processed for different fatty acid using gas chromatography (GC) with flame ionization detection. The neurosphere solution (80% Methanol) was transferred into a screw-cap glass vial and dried in a speed vac. After drying, methanol containing 14% boron trifluoride was added and the vials were briefly vortexed and heated in a hot bath at 100 °C for 10 min. After cooling, hexane and HPLC grade water were added sequentially. The vials were recapped, vortexed, and centrifuged to separate layers. An aliquot of the hexane layer was transferred to a GC vial and processed. Fatty acid composition was expressed as a percent of total identified fatty acids. The abundance of different fatty acid was quantified using Microsoft Excel and plotted using GraphPad Prism software.

##### Antibodies for Neurosphere Experiments

Western Blot: mouse anti-αSyn (Syn1 Clone 42,BD transduction labs, 610,787, 1:500 WB); rabbit anti-pS129 αSyn (MJFR13, Abcam 168,381, 1:500 WB); rabbit anti-vinculin (clone E1E9V, Cell Signaling Technologies 13,901, 1:1000 WB). Secondary Antibodies for Western Blot were as follows: LiCor IRDye 800CW – Dk α Ms (926–32,212) 1:5000; LiCor IRDye 800CW – Donkey anti-Rabbit (926–32,213) 1:5000; LiCor IRDye 680RD – Donkey anti-Mouse (926–68,072) 1:10,000; LiCor IRDye 800RD–Donkey-anti-Rabbit (926–68,073) 1:10,000.

Immunohistochemistry: Rabbit anti-VGLUT2 (Synaptic Systems Cat # 135 403, 1:500 IHC); Chicken anti-MAP2 (Biolegend Cat # 822,501, 1:1000 IHC); Mouse anti-GAD1/67 (Millipore Sigma Cat # MAP5406, 1:500 IHC); Rabbit anti-PAX6 (Invitrogen Cat # 426,600, 1:200 IHC); Mouse anti-S100β (Sigma, Cat # S2532, 1:500 IHC).

Secondary antibodies for IHC were as follows: Hoechst: Hoechst 33,342 Solution (20 mM) Cat #:62,249 (1:1000). Goat anti-Rabbit IgG (H + L) Highly Cross-Adsorbed Secondary Antibody, Alexa Fluor 488 (ThermoFisher A11034); goat anti-Chicken IgY (H + L) Secondary Antibody, Alexa Fluor 555 (ThermoFisher A21437); goat anti-Mouse IgG (H + L) Highly Cross-Adsorbed Secondary Antibody, Alexa Fluor 647 (ThermoFisher A21236).

#### Experimental Animals and YTX-7739 Oral Treatment

For the study, we used male 3K αSyn mutant mice (3K, line #3817) and expression-matched human WT αSyn transgenic mice (WT, line #3877) with two–threefold overexpression of human αSyn controlled by the Thy1.2 promoter (generation and characterization were previously described [13]. All mice were bred and maintained at the Hale BTM facility in accordance with National Institutes of Health guidelines on use of laboratory animals and an approved protocol by the Brigham and Women’s Hospital. Mice were kept in normal 12 h light/12 h dark cycles and had free access to food and water. For YTX-7739 treatment, 8-week (wk)-old non-transgenic, 3K and WT αSyn male transgenic mice were administered the compound accessible ad libitum in standard diet (Research Diets Inc, New Brunswick, NY).

#### Sequential Tissue Extractions of Mouse Brain Tissue

Mice were anesthetized, transcardially flushed with cold PBS, decapitated and the brains dissected on a chilled stage. Sequential extractions were performed as described [62]. Briefly, small pieces of cortex were homogenized via sonication at 4 °C in 2 volumes of TBS + [50 mM Tris–HCl, pH 7.4, 175 mM NaCl; 5 mM EDTA; protease inhibitor cocktail (Calbiochem, CA)] and spun for 45 min at 120,000 g. The pellet was subsequently sonicated in RIPA buffer (TBS +, 1% NP-40, 0.5% sodium deoxycholate, 0.1% sodium dodecyl sulfate) and incubated for 15 min, followed by ultracentrifugation for 30 min at 120,000 g. For quantifying pS129 deposits, the detergent-insoluble pellet was solubilized in 8 M Urea, 5% SDS [13, 62].

#### Intact-cell Cross-linking of Brain Tissue

Dissected cortical brain bits were gently minced with a razor blade, washed and re-suspended in PBS with EDTA-free complete protease inhibitors (Roche Applied Science). Intact-cell cross-linking was then conducted as previously described [13]. Briefly, the cell-permeable cross-linker DSG was prepared at 1 mM final concentration in DMSO immediately before use. Samples were incubated with DSG for 30 min at 37 °C with rotation. The reaction was quenched by adding Tris, pH 7.6, at 100 mM final concentration and incubated for 5 min at RT with rotation. After quenching and aspiration of the supernatant, cells were lysed in TBS containing 1% Triton X-100. Proteins were separated from cell debris by ultracentrifugation for 45 min at 120,000 g.

#### Western Blot Analyses of Brain Protein Lysates

For western blotting, 8–15 μg total protein of sequential extracts was each run on 4–12% Bis–Tris gels (Invitrogen, Carlsbad, CA) and electroblotted onto nitrocellulose membranes (Millipore, Bedford, MA). All cross-linked samples were blotted on PVDF membranes for enhanced retention of proteins. For improved immunodetection of αSyn (monomers of which are prone to washing off filters [63, 64], the membranes were fixed in 4% paraformaldehyde (PFA) for 10 min. After washing in PBST (phosphate-buffered saline with 0.2% Tween-20), membranes were blocked for 1 h at RT in PBS containing 5% bovine serum albumin (BSA). Blots were then incubated with hu anti-αSyn 15G7 (1:1000; Enzo Life Sciences), pS129 αSyn (51253, 1:500; Abcam), GCase (G4171; 1:1000; Sigma) or cathepsin B (RD Biosciences; 1:1000). Blots with cross-linked samples were incubated with syn1 (Mc42, 1:2000, BD biosciences). All abs were diluted in PBS containing 5% BSA overnight. After washing with PBST, membranes were probed with appropriate secondary antibodies (1:3000, American Qualex, CA), visualized with enhanced chemiluminescence (ECL, PerkinElmer, Boston, MA), and analyzed with the VersaDoc gel imaging system. Proteins were normalized to β-actin (A5441, Sigma; 1:5000), which was used as a loading control. DJ-1 was used as a quality control for cross-linking. Quantification of signal intensities was performed as described [40].

##### Immunohistochemistry

Diaminobenzidine, PK digestion, and fluorescence labeling was performed as previously described [13]. Briefly, sections were blocked in 10% normal serum and incubated overnight at 4 °C with antibodies to total LAMP1 (25,245, 1:500; Abcam), anti-pS129 αSyn (51253, 1:4000; Abcam) and tyrosine hydroxylase (AB 152; 1:1000, Millipore). This was followed by incubation with the appropriate fluorophore-conjugated secondary antibodies (1:500 in PBS; Alexa 488, 568, 647) for 3 h at RT. Nile Red (NRed; ab228553; abcam) was diluted 1:500.000, applied for 15 min and washed 5 × 10 min at the final staining step prior embedding with DAPI-containing mounting medium (Vectashield). Confocal microscopy was conducted with an Axiovert 35 microscope (Zeiss) mounted on a MRC1024 laser scanning confocal microscope (Bio-Rad), and each image was color-balanced. /Image analyses were done using Fiji ImageJ Software (NIH). The FIJI plug-in “colocalization highlighter” created a mask of overlapped pixels. The number or sizes or % area of the co-localized pixels on the resultant 8-bit images were quantified using the analyse particle function plugin. The FIJI plug-in “Maxima” was used to select NileRed puncta and the color spectrum of merged images (pS129 and Nile Red) shown in histograms and the ‘median cut’ saturation and frequency of the Nile Red LUT (all plugins of Fiji) were quantified of each 10 aggregates per cohort.

#### Behavioral Test

Mice were tested for their pole and rotarod performances as previously described (65). Briefly, for *pole testing*, mice were placed with the head oriented toward the top of a 50 cm vertical threaded metal pole with a diameter of 1 cm. Mice were timed as they descended to the base of the pole as a way assessing their ability to grasp and maneuver on a pole. The timing began as soon as mice oriented themselves downward. A maximum duration time of 1 min was set to avoid exhaustion. The mice were tested for 3 consecutive trials (climbing down to the base), and average times to ‘climb down’ were calculated for each mouse.

Motor coordination and motor skill learning were evaluated using an accelerating rotarod (Ugo Basile), and time spent on the rod was recorded. The first two days consisted of a habituation trial at constant speed [4 rpm for 5 min], followed by two trials of 4–40 rpm progressive acceleration within 5 min for 5 consecutive days, including 2 trials each day. On the next two days, the mice were tested only on the accelerating trials (4–40 rpm, 5 min). An inter-trial pause of at least 1 h was applied to avoid fatigue and stress, and a maximum cutoff of 5 min was used. Motor coordination was measured by mean latency to fall over the 3 consecutive days between groups.

#### Experimental Mouse Treatment Design, Quantifications, and Statistical Analysis

Experimental details specific for behavioral testing are included in behavior testing (see above). Details regarding each statistical test, biological sample size (n), and *p* value can be found in the corresponding figure legends and result section. All data are represented as mean ± SEM. SEM represents variance within a group. In all experiments, the genotypes can be found in the corresponding legends. Data were collected and processed side by side in randomized order for all experiments. Behavioral and histological tests were routinely performed blind to the conditions of the experiments. Unpaired, two-tailed *t* tests were used for comparison between two groups, with p ≤ 0.05 considered significant. For all comparisons involving multiple variables, ANOVA was performed followed by post hoc Tukey test for paired comparisons using p ≤ 0.05 for significance. Bar graphs show statistical information (mean ± SEM). All statistical analyses were performed using GraphPad Prism.

## Supplementary Information

Below is the link to the electronic supplementary material.

Supplementary file1 (DOCX 4609 KB)

Supplementary file2 (PDF 436 KB)

Supplementary file3 (PDF 442 KB)

Supplementary file4 (PDF 437 KB)

Supplementary file5 (PDF 437 KB)

Supplementary file6 (PDF 527 KB)

Supplementary file7 (PDF 509 KB)

Supplementary file8 (PDF 435 KB)

Supplementary file9 (PDF 597 KB)

Supplementary file10 (PDF 509 KB)

Supplementary file11 (PDF 1213 KB)

Supplementary file12 (PDF 946 KB)

Supplementary file13 (PDF 1046 KB)

Supplementary file14 (PDF 219 KB)

Supplementary file15 (PDF 517 KB)

Supplementary file16 (PDF 10402 KB)

Supplementary file17 (PDF 1213 KB)

Supplementary file18 (PDF 1200 KB)
