# Correction to: A Brain-Penetrant Stearoyl-CoA Desaturase Inhibitor Reverses α-Synuclein Toxicity

**DOI:** 10.1007/s13311-022-01268-x

**Published:** 2022-07-06

**Authors:** Silke Nuber, Chee Yeun Chung, Daniel F. Tardiff, Pascal A. Bechade, Thomas D. McCaffery, Kazuma Shimanaka, Jeonghoon Choi, Belle Chang, Waseem Raja, Esther Neves, Christopher Burke, Xin Jiang, Ping Xu, Vikram Khurana, Ulf Dettmer, Saranna Fanning, Kenneth J. Rhodes, Dennis J. Selkoe, Robert H. Scannevin

**Affiliations:** 1 Ann Romney Center for Neurologic Diseases, Department of Neurology, Brigham and Women Hospital and Harvard Medical School, 60 Fenwood Rd, Boston, MA 02115 US; 2 Yumanity Therapeutics, 40 Guest Street, Boston, MA 02135 US; 3 iNeuro Therapeutics, Cambridge, MA 02116 US; 4 Wave Life Sciences, 733 Concord Ave, Cambridge, MA 02138 US; 5 Verge Genomics, 2 Tower Pl, South San Francisco, CA 94080 US

## Correction to: Neurotherapeutics https://doi.org/10.1007/s13311-022-01199-7

The surname of coauthor Esther Neves was misspelled in this article as originally published.

The original article has been corrected.

