# Supplementary material for: A Brain-Penetrant Stearoyl-CoA Desaturase Inhibitor Reverses α-Synuclein Toxicity
Source: Neurotherapeutics. 2022 Apr 20;19(3):1018–36. doi: 10.1007/s13311-022-01199-7 (PMC9294123; doi:10.1007/s13311-022-01199-7)
Supplement: Supplementary file 1 — Supplementary file1 (DOCX 4609 KB) [file 13311_2022_1199_MOESM1_ESM.docx]

**Supplementary Figure 1** (A) Workflow of iCell human neuron plating, transfection, imaging, and analysis. iCell neurons were plated onto 96-well plate and transfected at DIV7. Automated live imaging was started from DIV8 with 12-hour interval for 12 days. At the end of experiment, data were analyzed by CL-Quant software with the automated algorithm developed by Nikon. (B) Representative brightfield and fluorescence (mApple) image after transfection. Images were taken by the automated imaging system in BioStation CT. (C) Example of automated detection of cell bodies (red), neurite (yellow), and nodes (blue dot) using CL-Quant software. (D) M17D aSyn-3K-YFP stable cells were treated with YTX-7739 or DMSO vehicle. Brightfield and fluorescence images of the neurons treated with DMSO vehicle (indicated as 0 µM YTX-7739) and 10 µM YTX-7739 are shown. Inclusions containing aSyn-3K-YFP are observed in the 0 µM YTX-7739 fluorescence image. αSyn-3K-YFP inclusions in the cells treated with 10 µM YTX-7739 appear to have a lower intensity compared to 0 µM YTX-7739. αSyn-3K-YFP was imaged in the 488 channel while nuclei were stained with Hoechst and imaged in the 405 channel. (E) Quantification of the αSyn-3K-YFP inclusion intensity in YTX-7739-treated M17D αSyn-3K-YFP stable cells. Intensity of the αSyn-3K-YFP intensities is represented by the relative spot intensity. Cells were treated with YTX-7739 from the 0.01 µM to 10 µM as well as DMSO vehicle (0 µM YTX-7739). Live cells were counted as an indication for potential effects of compound treatment on cell proliferation or toxicity. All values were normalized to DMSO vehicle. Graph is means ± standard deviation from 5 separate experiments, n=14. (F) Fatty acid desaturation index for C16 and C18 species were measured for M17D αSyn-3K-YFP stable cells treated with a concentration range between 0.12 µM to 10 µM YTX-7739. The fatty acid desaturation index for palmitoleic acid was calculated as the ratio between C16:1n7 to C16:0 and for oleic acid using the ratio between C18:1n9 to C18:0. Graph is means ± standard deviation from 2 separate experiments, n=6. (G) M17D/αSyn-E46K cells were treated with YTX-7739 (10, 3.3, 1.1 µM) or DMSO vehicle for 48 h and subjected to WB for total αSyn (mAb Syn1), αSyn pS129 (pAb EPY1536) and GAPDH (loading control). pS129/total αSyn ratios were calculated and normalized to DMSO vehicle the average of which was set to 1. 3 independent experiments (N=3) were performed on 3 different days in 3 or 4 independent wells (n=3,3,4; total n=10). (H) Fatty acid desaturation index for C16 and C18 species were measured for iGluta neurons treated with a concentration range between 0.37 µM to 10 µM of YTX-7739. Representative Western blot and quantifications (graphs are means ± STDEV). **, p<0.01, ***, p<0.001. One-way ANOVA plus Dunnett’s multiple comparisons test.

**Supplementary Figure 2 (A)** A seeding size of 20,000 cells/well for both the Corr and A53T line resulted in similarly sized neurospheres (600 – 900 μm) at day 60. **(B)** qPCR results of 3 batches of A53T and Corr cortical neurospheres. The delta CT value is the difference between the housekeeping gene CT value (GAPDH) and the CT value of the gene of interest. These normalized values indicate which genes are highly expressed and which genes are lowly expressed. A lower CT value (in red) indicates high gene expression, while a high CT value (in blue) indicates low gene expression. Four genes of interest: Map2 (Neuronal Marker), Synapsin I (Neuronal Maturity Marker), Pax6 (NSC Marker), and S100b (Astrocyte Marker).  **(C)** Immunocytochemistry of cryosectioned neurospheres shows the expression of neuronal marker MAP2, astrocyte marker S100B, NSC marker PAX6, and nuclear stain DAPI in the Corr neurospheres.  **(D)** Immunocytochemistry of cryosectioned neurospheres shows the expression of neuronal marker MAP2, nuclear stain Hoechst, glutamatergic neuron marker VGLUT2 and GABAergic neuron marker, GAD1/67, neuronal marker MAP2 and nuclear stain DAPI in Corr neurospheres. **(E-F)** Total levels of the essential fatty acids, C18:2n6 (linoleic acid) and C18:3n6 (γ-linolenic acid) in neurospheres. The analysis was conducted using one-way ANOVA with Tukey’s test. Data are mean + SD. **P<0.01; ***P<0.005; ****P<0.0001
